# *In silico *gene expression analysis – an overview

**DOI:** 10.1186/1476-4598-6-50

**Published:** 2007-08-07

**Authors:** David Murray, Peter Doran, Padraic MacMathuna, Alan C Moss

**Affiliations:** 1 General Clinical Research Unit, UCD School of Medicine and Medical Sciences, Mater Misericordiae University Hospital, Dublin 7, Ireland; 2 Gastrointestinal Unit, Mater Misericordiae University Hospital, Dublin 7, Ireland; 3 Division of Gastroenterology, Beth Israel Deaconess Medical Center, 330 Brookline Ave, Boston, MA 02215, USA

## Abstract

Efforts aimed at deciphering the molecular basis of complex disease are underpinned by the availability of high throughput strategies for the identification of biomolecules that drive the disease process. The completion of the human genome-sequencing project, coupled to major technological developments, has afforded investigators myriad opportunities for multidimensional analysis of biological systems. Nowhere has this research explosion been more evident than in the field of transcriptomics. Affordable access and availability to the technology that supports such investigations has led to a significant increase in the amount of data generated. As most biological distinctions are now observed at a genomic level, a large amount of expression information is now openly available *via *public databases. Furthermore, numerous computational based methods have been developed to harness the power of these data. In this review we provide a brief overview of *in silico *methodologies for the analysis of differential gene expression such as Serial Analysis of Gene Expression and Digital Differential Display. The performance of these strategies, at both an operational and result/output level is assessed and compared. The key considerations that must be made when completing an *in silico *expression analysis are also presented as a roadmap to facilitate biologists. Furthermore, to highlight the importance of these *in silico *methodologies in contemporary biomedical research, examples of current studies using these approaches are discussed. The overriding goal of this review is to present the scientific community with a critical overview of these strategies, so that they can be effectively added to the tool box of biomedical researchers focused on identifying the molecular mechanisms of disease.

## 1. Background

Investigations aimed at deciphering the molecular events that underpin the initiation and progression of disease are primarily targeted towards the profiling of biomolecules, whose aberrant expression, contributes to alterations in cellular function and ultimately lead to disease. By focusing on the mechanisms of disease, biomedical researchers aim to identify critical molecular events that can be targeted with novel therapeutic strategies. Thus, a key starting point in mechanisms of disease research is deciding how to identify these disease-associated biomolecules.

Historically such investigations focused on the characterisation of single molecules and studying their role in disease. The inherent weakness of such focused disease research strategies lies in the fact that complex diseases are usually polygenic and single molecule studies will not provide insights into the orchestrated response of a cell as it evolves within a diseased tissue. Thus, it is accepted that an overall view of the biomolecular composition of diseased tissue provides extraordinary opportunities to observe the global molecular response to disease. By visualising the entire response, researchers begin to understand the complex inter-relationships between biomolecules that contribute to changes in cell phenotype, and ultimately disease. A significant hurdle for biomedical researchers to overcome in the past has been how to access and analyse molecular information at such a detailed level. The answer to this has been the development of novel experimental and analytical methodologies that have, in many ways, redefined the biologists' toolkit.

A major enabling factor in molecular analysis of disease has been the recent completion of the human genome project. This landmark project has detailed and defined our genetic make-up provides all the information needed to understand both health and disease. Although greeted with much fanfare the completion of the genome-sequencing project is best seen as a new beginning for biomedical research, as the sequence merely lists our genetic composition and does not interpret the relevance of the information in health and disease. However the availability of this data coupled with ongoing sequence determination initiatives has provided a huge repository of sequence data for use in assembly projects and also for enabling continued developments in human transcriptomics, thus facilitating investigations of biological and disease mechanisms to be carried out on a genome wide scale.

All biological events in the cell are governed primarily by changes in the expression of key genes. The ability of a cell to switch on and off gene expression drives all biological function and activity. Gene transcription is crucial in normal events such as cell division, proliferation, differentiation and cell death. Conversely, gene transcription is a facilitator of the pathogenomic events that drive the development and progression of disease, as well as governing response to therapy. Much interest is therefore focused on the delineation of gene expression profiles to identify those key genes and gene clusters whose expression is altered in disease states. Research into the mechanism of diseases is underpinned by identifying these gene alteration patterns. By comparing gene expression profiles under different conditions, individual genes or groups of genes can be identified that play a key role in particular signalling cascades or particular cellular process or in disease aetiology. Expression profiling is also important for understanding gene functions and identifying therapeutic targets. Gene expression profiling is also crucial to identifying diagnostic, prognostic and predictive markers of disease. Effective methods are therefore required that can compare the expression of many genes within one tissue type and also to as compare the expression of one gene in various tissues or disease types.

Thus, biomedical researchers are equipped with both the map of the genome and an understanding of how gene expression events contribute to health and disease. However, to truly capitalise on this wealth of information, novel tools are required to permit identification of what genes are activated and suppressed in disease. Techniques capable of quantifying gene expression enable the development of our understanding of the distribution and regulation of gene products in normal and abnormal cell types. These include a variety of microarray and Serial Analysis of Gene Expression (SAGE) techniques, all of which have the ability to quickly and efficiently survey genome-wide transcript expression. The development of microarrays has improved our ability to simultaneously study the expression of many genes in a particular tissue. However there are also opportunities to exploit computational methodologies that profile expression of all genes, not just known genes on chips, in a quantitative and straightforward way. The availability of vast amounts of sequence data, coupled to advances in computational biology provides an ideal framework for *in silico *gene expression analysis. The last two decades have seen tremendous advances in computational approaches to understanding the molecular basis of disease, advances that have heralded a new era in biomedical research. The exponential growth of biologically relevant datasets has transformed the biological and biomedical research enterprise from a very data light to an information-heavy pursuit. This growth in available information has been matched by advances in our ability to understand and mine this new information. Biologists now routinely analyse huge microarray datasets, recreate biological networks, identifying protein folding patterns and model whole cell activity using computational strategies. All these advances are driven by computational strategies that match the availability of data, with the clear goal of identifying biologically relevant patterns in data. Indeed these technologies have been used to investigate the molecular events underpinning various malignancies, including breast, colon, lung, ovarian, pancreatic and prostate cancers [1]. In this review a number of these strategies and their important, emerging roles in disease research are discussed.

## 2. The assembly and organisation of *in silico *gene expression data

The growth in the number of EST mining projects is due mostly to the public availability of transcribed sequences. However, one of the major problematic issues associated with such gene-mining analysis is the high level of redundancy found among these sequences. Because a single gene may be expressed as mRNA many times, EST libraries may contain many identical or similar copies of the same EST derived from this mRNA. This overlap means that when one searches for a particular EST, they may retrieve a list of tags, many of which may represent the same gene. The development of Unigene began as an effort to eradicate such redundancy problems associated with EST analysis and also to establish a consensus regarding protocols among analysts. Maintained by the National Centre for Biotechnology Information (NCBI), Unigene is an automated analytical system for producing an organized view of the transcriptome. Unigene also addresses issues such as normalisation of data to allow the representation of rare transcripts by reducing the abundance of highly expressed genes [2].

High throughput cDNA sequencing is used to obtain EST sequences based on a study described in 1991, whereby cDNA clones were chosen at random and sequenced from one or both ends of their inserts [3]. The term EST was introduced to refer to this type of sequence, characterized by its short length (400–600 bases). ESTs are therefore powerful in the search for known genes because they greatly reduce the time required to locate a gene. Unigene partitions EST sequences into a non-redundant cluster, where each cluster represents a unique gene. The number of EST sequencing projects has grown and continue to grow [4-6] and the organisation of ESTs has allowed searching them to be used as an established and successful gene discovery tool in disease research [7,8].

An EST depository, dbEST, was developed as part of Genbank, the NIH sequencing database [9,10]. dbEST addresses the increasing amount of EST data being generated. Furthermore, a large amount of ESTs deposited into Genbank originate from the Cancer Genome Anatomy Project (CGAP), a collaborative network dedicated to deciphering the genetic changes that occur during the initiation and progression of cancer [11,12]. Because of the efforts of CGAP, a large variety of normal and transformed tissues are represented in the Unigene database including 117 different cancerous and 13 different precancerous cell types. The power of these libraries lies in their ability to allow the evaluation of the expression patterns of thousands of genes in a quantitative way without prior sequence information.

**Figure 1:**
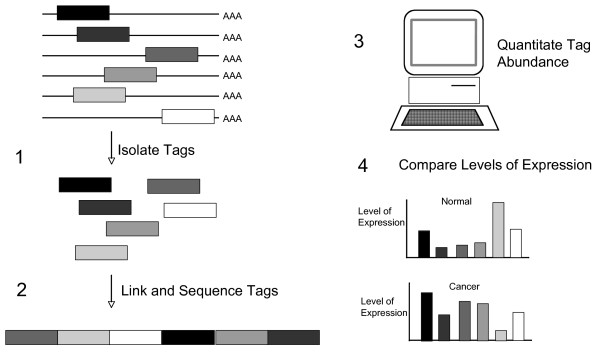
An overview of the SAGE process. The SAGE method for the comprehensive analysis of gene expression patterns consists of the following steps; **1**. SAGE tags containing sufficient information to uniquely identify a transcript are isolated by amplification; **2**. Tags are then linked and sequenced; **3**. The resulting sequence data are analyzed to identify each gene expressed in the sample and the levels at which each gene is expressed; **4**. This information forms a library that can be used to compare gene expression between tissues or cell types. For a review see [14].

Serial Analysis of Gene Expression (SAGE) is a method with the ability to efficiently quantitate and compare large numbers of transcripts [13]. The following articles give detailed accounts of SAGE library construction [13,14]. By isolating only a portion of the cDNA transcript, which is known as a SAGE tag, 50,000 transcripts for a given tissue can be analysed at once. Thus allowing an expression profile for that particular tissue to be generated (Figure 1). Analysis is achieved by forming concatamers (DNA segments composed of repeated sequences linked end to end) of SAGE tags and subsequently sequencing up to 30 tags at once. The frequency of each tag in the concatenated sequence reflects the cellular abundance of the corresponding transcripts allowing statistically significant comparisons of expression levels between two populations to be made [15]. SAGE produces a digital output, a format that makes it easily comparable, thus SAGE libraries constructed in different laboratories at different times can be compared. SAGE libraries are therefore used to analyze the differences in gene expression between cells or tissues where the frequency of each SAGE tag directly reflects transcript abundance thus generating an accurate picture of gene expression at both a qualitative and the quantitative level.

The EST and SAGE libraries described above represent ideal tools for the investigation and identification of disease related gene expression. These resources can be used in a wide range of applications, for example to identify genes of importance in disease or to analyze the effect of drugs on cells, ultimately providing insights into the disease pathways. Nevertheless, the power of these libraries as a comprehensive and quantitative transcript profiling method relies on efficient computational tools for data generation, management and analysis. These libraries are currently being exploited to define the transcriptomes of various tissues and diseases and furthermore to analyse the differences between the gene expression patterns of diseased cells and their normal counterparts. Various examples of the application of these tools to biomedical research are described herein.

Finally, these methods in comparison with microarray analysis, require no initial laboratory work in terms of sample generation and therefore demand less time and effort. Furthermore in comparison with the relatively expensive microarray where the price of an analysis often limits the amount of samples analysed, most of these in silico approaches are free of charge.

## 3. Analysis of differential gene expression

This review serves as an introduction and critical overview of computational methods for gene discovery and their applications in disease research. A key area of this research involves attempts to define the population of genes that are differentially expressed in a diseased tissue or in models of the disease process. Knowledge of the identity of such transcripts provides a useful starting point in the search for the critical molecular events contributing to the disease. To this end, there is currently immense interest in methodologies that allow a snapshot of the genetic machinery at work during a pathological process to be taken. These methodologies include microarray or 'gene chip' analysis or those computational techniques discussed herein. Although both microarray and *in silico *approaches can be publicly accessed, this review will focus primarily on in silico SAGE and EST profiling techniques. As gene discovery techniques, the *in silico *methods discussed herein have the advantage over microarray analysis of being relatively inexpensive. No specialised hardware or lab reagents are required. This allows many more comparisons between many tissue types and tissue collections to be easily made. Furthermore, in an effort to integrate the abundance of data generated from these various sources, many open-source tools, have been developed to compare and integrate microarray data with *in silico *data.

### 3.1 *In silico *est profiling strategies

For the modern biologist, there are numerous computational strategies that can be employed to assay gene expression. Many of these are based on utilising collections of expressed sequence tags (ESTs), unique segments of cDNA with base sequences identical to at least part of the coding region of a gene [3]. Because a large number of ESTs from diverse organ- and disease-derived cDNA libraries are being deposited in different databases, EST libraries are therefore an ideal source for expression profiling since EST clone frequency is in principle, proportional to the corresponding gene's expression level in a given tissue [16,17]. This article reviews the many open-source online tools that have been developed to aid the handling, analysis and exchange of gene expression data in the public forum.

The aforementioned EST and SAGE data collections represent virtual goldmines of information for the modern biologist. Furthermore, these libraries are excellent starting points for disease-related gene discovery. For example, the EST database (dbEST) currently contains > 28 million public entries. Nevertheless, in any expression profiling experiment, be it *in vitro *or *in silico*, appropriate considerations need to be taken into account. These include the quantity and quality of RNA, where increasing these factors will invariably increase the yield of reliable and comprehensive experimental results. Concern over these issues is reduced by performing computational expression profiling and further careful *in silico *analysis can significantly reduce the amount of lab work required. Another caution worth considering is the source tissue. Many of these tools allow the user to select micro-dissected tissue as apposed to bulk tissue, therefore making the gene expression profile generated more specific. It is apparent from the amount of genomic information assembled in databases such as Unigene, that efficient tools are needed to mine these collections in search of meaningful information. To exploit this large amount of information, computer algorithms have been developed for the discovery of both novel genes [18] and genes with limited tissue distribution and/or disease-specific expression [19].

### 3.2 Sagemap

One of these algorithms, SAGEmap, is an online tool, specifically designed to interpret SAGE data [18]. SAGE data from any source may be submitted to this repository and SAGE data from a wide variety of sources may therefore be studied. SAGE data from both bulk tissues and cell lines from various species are collected in SAGEmap. Data is available on all SAGE data, including tissue type, depositor and any treatment the tissue has undergone. Comparisons of individual SAGEmap tag libraries can be performed to provide a list of differentially expressed tags in specific tissue libraries. SAGEmap is a user-friendly tool maintained by the NCBI and undergoes frequent updating. However, one weakness associated with SAGEmap is that tags with a count of one are excluded due to single-pass sequencing associated with tag production. This has little effect on tags with high abundance (i.e. more than one count) but can result in the loss of tags with counts of less than one. Nevertheless, such analysis involving tags with high counts enables differences of statistical significance to be easily identified.

### 3.3 X profiler

**Figure 2:**
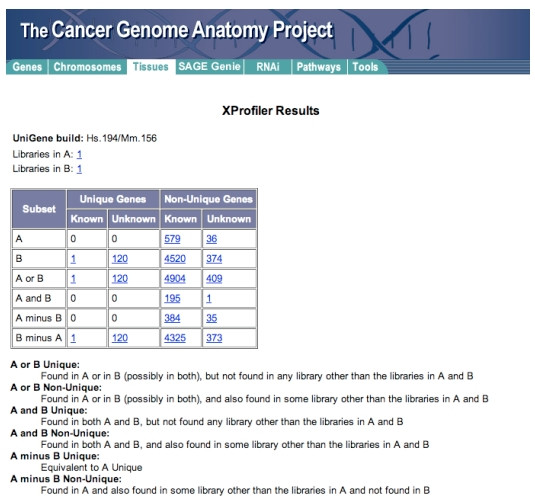
A typical output from the CGAP XProfiler online tool. In this example bulk breast cancer tissue is compared with normal tissue. This sample comparison was made on 21-October-2006.

The cDNA XProfiler is another tool that compares gene expression between two pools of libraries, where each pool can be a single library or a grouping of several libraries [21]. For example, a user may compare diseased lung tissue with healthy lung tissue or furthermore a user may compare two different types of diseased lung tissue. For a gene to be "present" in a library pool, there must be at least one EST sequence found in the UniGene cluster for that gene. XProfiler lists all the genes found in each pool and categorises them as unique or non-unique. XProfiler further classifies them into known or unknown genes. The results are finally tabulated to show how these genes are distributed between both pools (Figure 2). A typical X-profiler analysis proceeds as follows;

Firstly the user selects tissue types (organ of origin) for each pool. The user then decides what tissue preparation methodology they want to include for each pool (e.g. cell line, bulk tissue, microdissected etc.). The next step is to select the library protocol that is of interest (e.g. normalised libraries, subtracted libraries etc.). The user then decides what tissue histology is to be included in each pool (e.g. normal tissue in one pool and cancer tissue in another) and finally pools are named and the query is submitted.

X-Profiler populates the pools with libraries that correspond to the user requirements. Pools can then be reviewed and modified if necessary. Following review the pools are submitted for comparison.

The results page contains three sets of information:

• The UniGene Build number for this analysis.

• Links to each set of libraries.

• The gene expression comparison results in tabular form.

Gene Expression alterations are classified as unique or non-unique according to the pools of interest. The possible outputs for unique genes include.

First Pool Gene is only found in first pool.

Second Pool Gene is only found in second pool.

First Pool or Second Pool The total number of genes in both pools. These genes are found in either pools or maybe both, but not in any other library.

First Pool and Second Pool Genes found in both pools, but not in any other library.

In this way it is possible to identify all those genes whose expression is significantly changed between both pools (e.g. normal versus cancerous bulk breast tissue as separate pools therefore identifying genes that are altered in breast cancer). Figure 2 shows results obtained from this example comparison.

### 3.4 Digital gene expression displayer

Digital Gene Expression Displayer (DGED) is a tool for the comparison of gene expression between two pools of libraries. It can be used to compare either cDNA libraries or SAGE tag libraries. In contrast to XProfiler, it treats the presence of a gene in a library pool as a matter of degree. It compares the amount of a gene in one pool with the amount of the same gene in another pool. This comparison is reduced to two numbers: the sequence odds ratio and measure of significance. The formula used in DGED to calculate the sequence odds ratio between two pools A and B is; (Sequences in A/Total Sequences in pool A)/(Sequences in B/Total Sequences in pool B). DGED results are ordered by this odds ratio, with all cases of "NaN" (not a number) topping the list. NaN occurs when the denominator of the equation is 0, i.e., there are no sequences of a gene in pool B. An advantage cDNA DGED has many over other *in silico *gene expression techniques is that the user may select microdissected tissue source over bulk tissue thus giving a more specific gene expression output [22]. Another strength of DGED is that unlike the cDNA xProfiler, which lists every gene (even if an EST is seen only once in a pool) in both groups, the DGED finds only the statistically significant differences, based on the sequence odds ratio and a Bayesian test.

### 3.5 Digital differential display

Digital Differential Display (DDD) is a powerful web-based bioinformatic tool for the identification of differential gene expression. DDD uses the EST profiles of normal and disease cDNA libraries represented in the NCBI UniGene database. DDD compares the number of assignments of ESTs from different libraries, or pools of libraries to a specific UniGene cluster [1,23,24]. Fishers' exact test is used to restrict the output to statistically significant differences (P ≤ 0.05). It is therefore straightforward for users to then omit non-significant results from subsequent analysis. The output from DDD provides a numerical value denoting the fraction of sequences from each pool that maps to a specific cluster [25]. An example of a DDD experiment proceeds as follows;

#### 3.5.1 Description of ddd experiment

**Figure 3:**
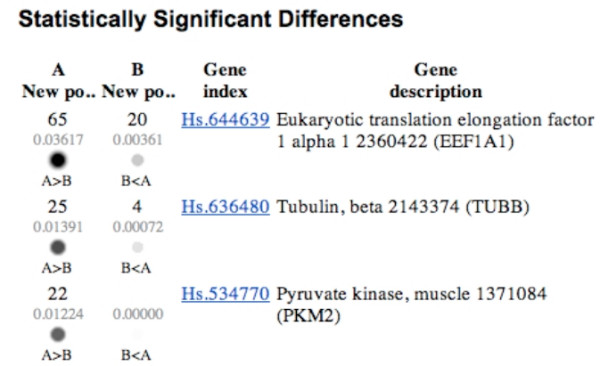
Typical DDD output. Following the selection of pools (A and B) for comparison, statistically significant differences are represented. Each line represents a gene. For each gene, the numbers represent the number of times that gene is represented in that particular pool. The p value for the difference is presented below that figure. Information on the gene, including its name, abbreviated title and unigene number are also presented.

In a typical DDD experiment the user must select which tissue libraries are to be assinged to each pool. The pools will then be compared. DDD compares the EST constituents of various tisue types, depending on which libraries are selected thereby determining the relative representation of each sequence in the libraries being compared. The DDD output is in the form of a web file that has links to Unigene clusters that correspond to the EST's that are differentially expressed between the two tissues (Figure 3).

DDD uses Fisher's extract test to restrict the output to statistically significant differences (*P *≤ 0.05) therby determining the statistical significance of the number of times sequences from the selected libraries are assigned to a specific UniGene cluster. This is a statistical test for analyzing categorical data when the sample sizes are small. It has been argued that Fisher's exact test is too conservative, and is not appropriate to a setting where the total number of data values in the contingency table is not fixed [26]. The Bayesian methods used in xProfiler have a less conservative statistical threshold. The analysis is also restricted so that genes with over 1000 sequences in UniGene are included. A limitation of DDD is that these requirements place limitations on the capabilities of the analysis. Unless there are a large number of sequences in each pool, the frequencies of genes are generally not found to be statistically significant. Furthermore, the wide variety of tissue types, cell types, histology, and methods of generating the libraries can make it difficult to attribute significant differences to any one aspect of the libraries.

### 3.6 Digital extractor

A major limitation of *in silico *gene mining approaches is the cumbersome nature of the subsequent data analysis. The output from DDD is a list of Unigene clusters representing known genes, and sequences without homology to known genes that are significantly altered between selected tissue libraries. To expedite this strategy the data derived from the DDD comparisons can be processed using Digital Extractor. This application provides for high throughput processing of DDD output, by performing automated annotation of the output clusters. Digital Extractor can be used to both compile the profiles of known genes differentially expressed and also to annotate those clusters containing cDNAs without homology to known genes [27]. It utilises Contig Assembly Program-3 (CAP3) for assembly of EST clusters, Repeat Masker to mask repetitive elements and BLAST for gene identification [27].

### 3.7 How these methods compare

As is evident from the previous sections, there are a variety of algorithms available to mine open source gene expression data. Table 1 provides a summary of the various tools and websites described herein while Table 2 lists their strengths and weaknesses. Given the variation in library compilation, tag format (EST or SAGE), statistical threshold, and data output associated with each method, they are unsuitable for direct comparisons in analysis of specific expression profiles. In general, DDD and cDNA DGED probably demonstrate most utility in terms of number of libraries, tissue descriptions, and detailed results output. It must be stressed that many of these tools are subject to frequent updating and improvement. Nevertheless, individual user preference is an apparent factor in deciding which tool to apply. For example, a quick search of the literature reveals that different investigators are successfully employing the different tools outlined in this review. Despite early hopes that such methods would provide automated deciphering of transcriptional profiles, it has become clear that supplementary experimental insight is required to validate computational "discoveries".

**Table 1:** Summary of *in silico *gene expression tools

Resource	Application	Web Address
CGAP	Online genetics resource for cancer researchers including online analytical tools.	
DDD	Online EST comparison.	
DGED	Online identification of significantly different gene expression	
GENBANK	DNA, RNA & protein sequence database	
SAGEmap	Resource for the analysis of SAGE data.	
UniGene	A database of the transcriptome. Organises transcripts into specific clusters.	
XProfiler	Compares gene expression between two pools of libraries	

**Table 2:** A comparison of the strengths and weaknesses of in silico gene expression mining tools

DDD
Strengths:
Size of EST databases in Unigene
Conservative test (Fisher's exact test) used to determine significance
Absolute and relative counts given

Weakness:
Libraries with low EST count excluded by analysis
Limited number of "normal tissue" libraries

DGED

Strengths:
Statistically parameters can be varied
Results linked to tissue microarray data
Ability to select origin/type of tissue (e.g. micro dissected etc).
Genes with low abundance included

Weakness:
Comparison based on odds ratio

Sagemap

Strengths:
Wide variety in the source of SAGE data available.
Accounts for differences in sample size between groups

Weakness:
Exclusion of tags with low counts

XProfiler

Strengths:
Ability to compare groups and pools of libraries.
Outputs genes as unique/non-unique and known/unknown.
Ability to select origin/type of tissue (e.g. micro dissected etc).

Weakness:
Exclusion of tags with low counts

Common Strengths:
Freely available via internet
Unbiased view of transcriptome

Common Weaknesses:
Reliability of initial sequencing experiments.
Limited background knowledge of original tissues
Significant false positive rate/false negative rate unknown

## 4. Applications in biomedical research

The rapid expansion of nucleotide sequence data available in public databases has revolutionised biomedical research. The growth of nucleotide sequence databases has made 'virtual' or electronic profiling of gene expression routine. For the purpose of this review, examples of the applications of computational methods will be refined mostly to cancer research projects.

### 4.1 Gene expression in health and disease

Homeostasis in healthy tissue is dependent on the expression of genes that ensure cells have the machinery to deal with everyday events and furthermore ensure our well-being. However, unchecked variations in gene expression levels in a cell often lead to the initiation and progression of a disease process, such as cancer. It is therefore a propriety in modern biomedical research to determine and compare what genes are turned on and off in disease tissue and normal tissue. Using the approaches described herein, groups of genes that are characteristic of disease and may also be driving the disease process can be identified. Such genes may furthermore provide attractive targets for novel therapies in our efforts to overcome these debilitating diseases. Due to the extensive information obtained from genome sequencing, many of these techniques output ESTs of known and unknown genes. It is therefore dependent on the individual user whether known or novel genes take priority for further studies.

### 4.2 Cancer-associated genes

Cancer is a genetic disease. Expression profiling, as a powerful genomic tool, holds great promise in cancer molecular medicine and cancer research. This is because cancer is a complex polygeneic and multifactorial disease, resulting from successive changes in the genome of cells and from the accumulation of molecular alterations in both tumour and host cells [28]. Such genetic alterations effect regulatory pathways and cellular processes such as proliferation, differentiation, cell cycle, DNA repair and apoptosis and can also lead to genetic instability, tumourigenesis, malignancy, and an invasive and drug-resistant phenotype. Therefore, an understanding of the molecular behaviour of tumours would aid their molecular classification and also aid the decision-making regarding therapeutic approaches [29].

Computational methods of transcriptional profiling have been applied to further the understanding of all aspects of cancer biology. For example the identification of highly expressed genes may provide significant information thereby enhancing our understanding of tumourigenesis or serve as biomarkers or prognostic markers of malignancy [24,25,30]. DDD has been used to investigate gene expression in a wide variety of cancers including breast, colon, lung, ovarian, pancreatic and prostate cancers [1]. In a study by Scheurle *et al *[1], these cancers were found to share similar expression profiles, a concept that was proven using other laboratory techniques such as RT-PCR. *In silico *methods have identified the kallikrein genes, KLK6 and KLK10 to be overexpressed in colorectal, pancreatic and ovarian cancers [24,31].

As apposed to simply identifying and compiling lists of genes, many studies have displayed genes identified *in silico *to be of functional importance as exemplified in two recent studies investigating gastric and colorectal cancer respectively [32,33]. In these studies, genes identified as being differentially expressed using DDD were confirmed to have importance in key aspects of tumour cell biology such as cell proliferation and invasion. In both studies altered gene expression was confirmed with PCR using *ex vivo *cancer tissue in comparison with normal tissue. RNAi was used to knockdown gene expression, which resulted in decreased tumour cell proliferation and invasion. DDD has recently been used to identify genes with promoter similarities and that are therefore co-regulated in colorectal cancer [34]. Similarly, genes downregulated in gastric cancer were recently identified using DGED. In this study by Yanglin *et al *[35] KCNE2, a downregulated gene, was identified as a novel gastric cancer associated gene. Furthermore, the functional importance of KCNE2 was highlighted whereby overexpression resulted in growth inhibition. A recent study has demonstrated the tissue specific gene expression of various tumour types [22]. Dennis *et al *[22] employed SAGE and DDD to display strict differences in the expression patterns of different tumour types that could be used as markers of the various tumour types and for a better assessment of patient prognosis and optimal, tailored therapy. The study by Dennis *et al *[22] also identified novel potential tumour markers. Lipophilin B was identified to have expression restricted to breast and ovarian cancers while glutathione peroxidase 2 was specifically enhanced in colon and pancreatic cancers.

The use of SAGE to identify genes associated with the latter stages of cancer has recently been displayed [36]. In a study by Shen *et al *[36] the expression of advanced breast cancer was compared with that of benign tissue libraries and to identify fifty-three differentially expressed genes to be correlated with breast adenocarcinoma, a subset of which were successfully confirmed by RT-PCR. Likewise, genes associated with breast metastasis have been identified using SAGE [37]. The role of estrogen in the progression of breast cancer has been elucidated using a SAGE approach [38]. SAGE analysis has recently been used to identify biomarkers of gastric cancer [39,40]. In a study by Yasui *et al *[37] SAGE was employed to identify differentially expressed genes in three categories; in gastric cancer in comparison to normal gastric mucosa, in advanced gastric cancer in comparison with early stage disease and in lymph node metastasis in comparison with primary tumours. A custom 395-element cDNA microarray representing these genes was then fabricated for use in diagnostics. The study by Yasui *et al *[39] represents the translational application of *in silico *gene expression profiling.

A recent study applied *in silico *EST profiling techniques to characterise various cancers including liver cancer where Bcl-x2 was identified as a novel liver cancer-associated gene [41]. Similarly, a combination of SAGE and microaray analysis has been employed to identify biomarkers of bladder cancer [42]. In this study by Wang *et al *[42] UCA1 was identified as a specific and sensitive biomarker of disease, which could be detected in the urine of bladder cancer patients. Another study utilised microarray analysis in conjunction with the *in silico *analysis of CGAP EST libraries to identify loss of annexin A1 expression in breast cancer [43]. This study and those studies outlined herein exhibit the value of *in silico *strategies in discovering biomarkers with clinical relevance in cancer detection and disease classification.

### 4.3 Tissue-specific gene expression

Gene expression profiling techniques can be applied to the identification of tissue specific transcripts or clusters of transcripts. Successful identification of those genes with specific expression in specific tissue types will aid our understanding of diseases arising in these sites. The large number of publicly available cDNA libraries corresponding to different tissues can be exploited using techniques such as DDD to identify genes with tissue specific expression. This approach has recently been employed to identify transcripts with preferential expression in renal podocytes [44]. This was the first study to use DDD to predict cell type specific gene expression. The authors successfully identified a protein SLM2 to be specifically expressed in renal podocytes and upregulated in proteinuric glomerular diseases and furthermore to be involved in VEGF alternative splicing. The study by Cohen *et al *[44] displays the potential that a gene discovery technique such as DDD has to predict and further our understanding of cell type specific gene expression.

## 5. Conclusion

Online open-source sequence data represents an excellent resource for identifying differential gene expression. Indeed, these resources are popular starting points in many disease gene discovery research programmes. These datasets are collected and annotated in highly organised online databases. The modern biomedical investigator therefore has the ability to genomically profile diseases or distinctions of interest thereby identifying differentially expressed genes. This article summarises the various tools available to mine these collections. Although many of these tools, and particularly their interfaces, are quite basic in design, they nevertheless represent an excellent resource for gene discovery. Furthermore, although these tools can act at good starting points in disease gene discovery there is a need for experimental validation of *in silico*-derived differential expression results.

EST and SAGE libraries are not without their limitations [45,46]. One limitation associated with the use of EST databases is that only highly expressed genes have been sampled adequately to provide sufficient corresponding EST counts for reliable molecular profiling. There is therefore a bias towards highly-expressed genes in libraries. Investigators must therefore be cognisant that expression profiles garnered from EST libraries may not contain these low abundance transcripts. As single-read sequences, ESTs are prone to sequencing error, although sequencing errors do not preclude identification of the original gene. Furthermore, the 5' ends of genes are underrepresented in EST databases. Libraries from which ESTs are derived can be contaminated with genomic material and using ESTs will not detect genes from tissues or cells, which are difficult to obtain mRNA from. ESTs omit introns which may contain important gene regulatory sequences [45]. SAGE libraries are subject to variable tag specificity, and the restriction enzymes used in tag generation yield fragments of various lengths [46].

Nevertheless, genes identified and observations made by EST library mining must be validated at a laboratory level either using *ex vivo *tissue or *in vitro *cell line models. Furthermore, as outlined herein, a majority of research groups using these approaches are also identifying the functional importance of these differentially expressed genes in the disease setting.

In conclusion the use of *in silico *gene mining strategies provides an excellent framework for the initial identification of key genes and gene clusters whose expression is altered in disease tissue. The data generated in these investigations provide a starting point for investigations aimed at delineating the molecular basis of disease.

## Abbreviations

SAGE: Serial Analysis of Gene Expression

DDD: Digital Differential Display

EST: Expressed Sequence Tag

NCBI: National Centre for Biotechnology Information

CGAP: Cancer Genome Anatomy Project.

## Competing interests

The author(s) declare that they have no competing interests.

## Authors' contributions

DM and AM drafted the manuscript.

PD conceived of the article and participated in the coordination and drafting of the manuscript.

PMM has been involved in the drafting of the manuscript.

All authors have made substantial contributions to the conception and layout design of this manuscript.

All authors have been involved in drafting the manuscript and revising it critically for important intellectual content.

All authors read and approved the final manuscript.
